# Supplementary figures and images for: Periodontitis, dental plaque, and atrial fibrillation in the Hamburg City Health Study
Source: PLoS One. 2021 Nov 22;16(11):e0259652. doi: 10.1371/journal.pone.0259652 (PMC8608306; doi:10.1371/journal.pone.0259652)

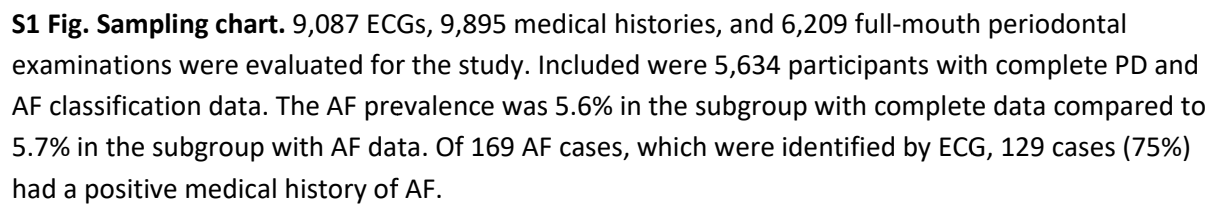

Supplement: S1 Fig — 9,087 ECGs, 9,895 medical histories, and 6,209 full-mouth periodontal examinations were evaluated for the study. Included were 5,634 participants with complete PD and AF classification data. The AF prevalence was 5.6% in the subgroup with complete data compared to 5.7% in the subgroup with AF data. Of 169 AF cases, which were identified by ECG, 129 cases (75%) had a positive medical history of AF. (PDF) [file pone.0259652.s001.pdf]
