# Supplementary material for: Periodontitis, dental plaque, and atrial fibrillation in the Hamburg City Health Study
Source: PLoS One. 2021 Nov 22;16(11):e0259652. doi: 10.1371/journal.pone.0259652 (PMC8608306; doi:10.1371/journal.pone.0259652)
Supplement: S1 Table — The potential association between PD and AF was studied in a low risk group. Participants with a history of hypertension, stroke, diabetes mellitus, heart failure, myocardial infarction, current smoking, and/or BMI above 35 (N = 4,890) were excluded. This resulted in 1,319 subjects, which included 18 cases of AF (AF prevalence: 1.36%). Shown are the results of the crude logistic regression model without any adjustments. (DOCX) [file pone.0259652.s002.docx]

**S1 Table. Association between periodontitis and atrial fibrillation in low risk subjects:
Crude logistic regression model**

| Model | Variable | Grades | Odds ratio per SD | 95% CI | p-value |
| --- | --- | --- | --- | --- | --- |
| Unadjusted | Periodontitis | none/mild | Ref |  |  |
|  |  | Moderate | 0.86 | 0.31-2.45 | 0.784 |
|  |  | Severe | 1.32 | 0.33-5.35 | 0.695 |

N = 1,319 out of 5,634 in the whole sample; 18 cases of AF

The potential association between PD and AF was studied in a low risk group. Participants with a history of hypertension, stroke, diabetes mellitus, heart failure, myocardial infarction, current smoking, and/or BMI above 35 (N = 4,890) were excluded. This resulted in 1,319 subjects, which included 18 cases of AF (AF prevalence: 1.36%). Shown are the results of the crude logistic regression model without any adjustments.
