# Supplementary material for: Periodontitis, dental plaque, and atrial fibrillation in the Hamburg City Health Study
Source: PLoS One. 2021 Nov 22;16(11):e0259652. doi: 10.1371/journal.pone.0259652 (PMC8608306; doi:10.1371/journal.pone.0259652)
Supplement: S2 Table — Age was strongly associated with AF, which corresponded with the results shown in the main paper for the whole sample of 5,634 participants. In contrast, sex was not associated with AF, which differed from the results obtained for the whole sample, in which women had lower odds of AF than men. (DOCX) [file pone.0259652.s003.docx]

**S2 Table. Association between periodontitis and atrial fibrillation in low risk subjects:
Multivariable logistic regression model**

| Model | Variable | Category | Odds ratio per SD | 95% CI | p-value |
| --- | --- | --- | --- | --- | --- |
| Multivariable | Periodontitis | none/mild | Ref |  |  |
|  |  | Moderate | 0.60 | 0.21-1.75 | 0.352 |
|  |  | Severe | 0.65 | 0.15-2.76 | 0.556 |
|  | ***Age*** |  | ***2.68*** | ***1.62-4.43*** | ***<0.001*** |
|  | Sex | Men | Ref |  |  |
|  |  | Women | 1.10 | 0.42-2.90 | 0.843 |

N = 1,319 out of 5,634 in the whole sample; 18 cases of AF.

Age was strongly associated with AF, which corresponded with the results shown in the main paper for the whole sample of 5,634 participants. In contrast, sex was not associated with AF, which differed from the results obtained for the whole sample, in which women had lower odds of AF than men.
