# Supplementary material for: Periodontitis, dental plaque, and atrial fibrillation in the Hamburg City Health Study
Source: PLoS One. 2021 Nov 22;16(11):e0259652. doi: 10.1371/journal.pone.0259652 (PMC8608306; doi:10.1371/journal.pone.0259652)
Supplement: S3 Table — In order to examine the association between PD and AF in a high risk group, subgroup analysis in participants with hypertension was performed. As shown in the Table, 3,844 participants had hypertension and the fraction of participants with severe PD was higher in hypertensive compared with normotensive participants. (DOCX) [file pone.0259652.s004.docx]

**S3 Table. Arterial hypertension and periodontitis severity grades**

|  | Normotensive | Hypertensive | p-value |
| --- | --- | --- | --- |
| N | 3228 | 6301 |  |
| Periodontitis (%) |  |  | <0.001 |
| none/mild | 633 (30.3) | 768 (20.0) |  |
| Moderate | 1150 (55.0) | 2266 (58.9) |  |
| Severe | 308 (14.7) | 810 (21.1) |  |

High risk group with complete data n = 3,844. AH: arterial hypertension

In order to examine the association between PD and AF in a high risk group, subgroup analysis in participants with hypertension was performed. As shown in the Table, 3,844 participants had hypertension and the fraction of participants with severe PD was higher in hypertensive compared with normotensive participants.
