# Supplementary material for: Periodontitis, dental plaque, and atrial fibrillation in the Hamburg City Health Study
Source: PLoS One. 2021 Nov 22;16(11):e0259652. doi: 10.1371/journal.pone.0259652 (PMC8608306; doi:10.1371/journal.pone.0259652)
Supplement: S4 Table — The Table shows the age- and sex adjusted multivariable logistic regression model for the high risk group. Age and sex were strongly associated with AF, which corresponded with the results shown in the main paper for the whole sample of 5,634 participants. However, no association between severe or moderate PD and AF could be observed. (DOCX) [file pone.0259652.s005.docx]

**S4 Table. Association between periodontitis and atrial fibrillation in hypertensive subjects:
Multivariable logistic regression model**

| Model | Variable | Units | Odds Ratio per SD | 95% CI | p-value |
| --- | --- | --- | --- | --- | --- |
| Unadjusted |  |  |  |  |  |
|  | Periodontitis | none/mild | Ref |  |  |
|  |  | moderate | 1.10 | [0.79;1.53] | 0.572 |
|  |  | severe | 1.36 | [0.93;1.98] | 0.113 |
| Multivariable age, sex |  |  |  |  |  |
|  | Periodontitis | none/mild | Ref |  |  |
|  |  | moderate | 0.89 | [0.63;1.24] | 0.486 |
|  |  | severe | 0.90 | [0.61;1.34] | 0.618 |
|  | Age |  | 2.40 | [2.04;2.81] | <0.001 |
|  | Sex | Men | Ref |  |  |
|  |  | Women | 0.63 | [0.48;0.81] | <0.001 |

The Table shows the age- and sex adjusted multivariable logistic regression model for the high risk group. Age and sex were strongly associated with AF, which corresponded with the results shown in the main paper for the whole sample of 5,634 participants. However, no association between severe or moderate PD and AF could be observed.
