# Supplementary material for: Periodontitis, dental plaque, and atrial fibrillation in the Hamburg City Health Study
Source: PLoS One. 2021 Nov 22;16(11):e0259652. doi: 10.1371/journal.pone.0259652 (PMC8608306; doi:10.1371/journal.pone.0259652)
Supplement: S5 Table — CRP, IL-6, and the odds of AF increased as a function of PD severity grades in non-adjusted analysis. (DOCX) [file pone.0259652.s006.docx]

**S5 Table. CRP and IL-6 plasma concentrations according to periodontitis grades**

|  |  | **Periodontitis** | | |  |
| --- | --- | --- | --- | --- | --- |
|  | **Total N=6,209** | **Non/mild** | **Moderate** | **Severe** | **p-value** |
| **hs-CRP µg/mL Median [IQR]** | 0.12 [0.06, 0.26] | 0.10 [0.06, 0.23] | 0.11 [0.06, 0.25] | 0.13 [0.07, 0.30] | ***<0.001*** |
| **IL-6 ng/L Median [IQR]** | 1.64 [1.18, 2.39] | 1.47 [1.03, 2.08] | 1.57 [1.16, 2.23] | 1.80 [1.34, 2.69] | ***<0.001*** |
| **AF OR [95% CI]** | - | Ref. | 1.31 [0.97-1.79]^1^ | 1.66 [1.17-2.38]^2^ | ^1^0.081 ***^2^0.005*** |

CRP, IL-6, and the odds of AF increased as a function of PD severity grades in non-adjusted analysis.
