# Supplementary material for: Periodontitis, dental plaque, and atrial fibrillation in the Hamburg City Health Study
Source: PLoS One. 2021 Nov 22;16(11):e0259652. doi: 10.1371/journal.pone.0259652 (PMC8608306; doi:10.1371/journal.pone.0259652)
Supplement: S6 Table — Exposure: Periodontitis, outcome: Atrial fibrillation. (DOCX) [file pone.0259652.s007.docx]

**S6 Table. Mediation analysis for IL-6 and CRP**

|  | **IL-6** | **p-value** | **CRP** | **p-value** |
| --- | --- | --- | --- | --- |
| **ACME** | 0.0027 | 0.202 | -0.0002 | 0.332 |
| **ADE** | 0.019 | 0.16 | -0.0144 | 0.046 |

ACME: average causal mediation effect; ADE: average direct effect

**Exposure: Periodontitis, outcome: Atrial fibrillation**
