# Supplementary material for: Periodontitis, dental plaque, and atrial fibrillation in the Hamburg City Health Study
Source: PLoS One. 2021 Nov 22;16(11):e0259652. doi: 10.1371/journal.pone.0259652 (PMC8608306; doi:10.1371/journal.pone.0259652)
Supplement: Attachment — Submitted filename: Response to Reviewers.docx [file pone.0259652.s008.docx]

**PONE-D-21-08970 – Reply to Editor’s comments**

Dear Editor,

thank you very much for your helpful comments. Here is our reply:

**Comment 1: “You may remove the patients with AF risk (i.e., HTN, DM, Stroke events, etc.) from the analyses. Then, you may see clear results without the statistical models.”**

**Reply:** We did this. Participants with a history of hypertension, stroke, diabetes mellitus, heart failure, myocardial infarction, current smoking, and/or BMI above 35 (N = 4,890) were excluded. This resulted in 1,319 subjects, which included 18 cases of AF (AF prevalence: 1.36%). Tab. 1 shows the results of the crude logistic regression model without any adjustments. The p-values were clearly above 0.05 and therefore an association between periodontitis (exposure) and AF (outcome) was unlikely. However, it should be noted that due to the low prevalence of AF in this low risk group, the power of this analysis to detect an association may have been insufficient (please, see our power calculation in the Methods section of our paper).

**Table 1: Association between periodontitis and atrial fibrillation in low risk subjects:
Crude logistic regression model**

| Model | Variable | Grades | Odds ratio per SD | 95% CI | p-value |
| --- | --- | --- | --- | --- | --- |
| Unadjusted | Periodontitis | none/mild | Ref |  |  |
|  |  | moderate | 0.86 | 0.31-2.45 | 0.784 |
|  |  | severe | 1.32 | 0.33-5.35 | 0.695 |

N = 1,319 out of 6,209 in the whole sample; 18 cases of AF

The age- and sex adjusted multivariable logistic regression model for the low risk group is shown in Tab. 2. It is obvious that age was strongly associated with AF as was also shown in our paper for the whole sample of 6,209 participants. In contrast, sex was not associated with AF. This differed from the results obtained for the whole sample of 6,209 participants, in which women had lower odds of AF than men. Whether or not this difference is real may however be questionable because of the low power of the subgroup analysis.

**Table 2: Association between periodontitis and atrial fibrillation in low risk subjects:
Multivariable logistic regression model**

| Model | Variable | Category | Odds ratio per SD | 95% CI | p-value |
| --- | --- | --- | --- | --- | --- |
| Multivariable | Periodontitis | none/mild | Ref |  |  |
|  |  | Moderate | 0.60 | 0.21-1.75 | 0.352 |
|  |  | Severe | 0.65 | 0.15-2.76 | 0.556 |
|  | ***Age*** |  | ***2.68*** | ***1.62-4.43*** | ***<0.001*** |
|  | Sex | Men | Ref |  |  |
|  |  | Women | 1.10 | 0.42-2.90 | 0.843 |

N = 1,319 out of 6,209 in the whole sample; 18 cases of AF

**Comment 2: “Please analyze h-CRP and IL-6 as well. If the inflammatory markers do not play a role in AF, what would be potential mechanism?”**

As shown in Tab. 3, CRP, IL-6, and the odds of AF increased as a function of periodontitis severity grades in non-adjusted analysis. Thus, it cannot be excluded that the inflammatory markers play a role in the mechanism of AF. The statistical models shown in Tab. 4 show that neither IL-6 nor CRP is associated with AF after adjusting for age and sex, whereas aging and male sex are strongly associated with AF. It is known that the plasma concentrations of CRP and IL-6 are higher in healthy individuals aged over 65 years compared to younger people [1] and that healthy women have higher CRP levels than healthy men [2]. On the other hand, aging and sex are linked to a vast number of additional effects and it is impossible to confirm the potential role of the inflammatory markers in AF on the basis of this cross-sectional study. Thus, your question cannot be answered at this stage.

**Table 3: CRP and IL-6 plasma concentrations according to periodontitis grades**

|  |  | **Periodontitis** | | |  |
| --- | --- | --- | --- | --- | --- |
|  | **Total N=6,209** | **Non/mild** | **Moderate** | **Severe** | **p-value** |
| hs-CRP µg/mL Median [IQR] | 0.12 [0.06, 0.26] | 0.10 [0.06, 0.23] | 0.11 [0.06, 0.25] | 0.13 [0.07, 0.30] | ***<0.001*** |
| IL-6 ng/L Median [IQR] | 1.64 [1.18, 2.39] | 1.47 [1.03, 2.08] | 1.57 [1.16, 2.23] | 1.80 [1.34, 2.69] | ***<0.001*** |
| AF OR [95% CI] | - | Ref. | 1.31 [0.97-1.79]^1^ | 1.66 [1.17-2.38]^2^ | ^1^0.081 ***^2^0.005*** |

We added the tables to our submission as supplementary tables uploaded as separate "supporting information" files. In addition, we added a paragraph to the Discussion section to discuss the impact of our results on the inflammatory hypothesis of AF. Furthermore, we made a number of other minor changes to improve the quality of our submission (please, see “manuscript with track changes”).

**Table 4: Logistic regression - outcome: Atrial fibrillation.**

| Model | Variable | Units | Odds Ratio per SD | CI 95 | p-value |
| --- | --- | --- | --- | --- | --- |
| Model 1 |  |  |  |  |  |
|  | Periodontitis | none/mild | Ref |  |  |
|  |  | moderate | 0.88 | 0.56-1.37 | 0.557 |
|  |  | severe | 0.81 | 0.48-1.37 | 0.434 |
|  | ***Age*** |  | ***2.71*** | ***2.18-3.36*** | ***<0.001*** |
|  | ***Sex*** | ***Men*** | ***Ref*** |  |  |
|  |  | ***Women*** | ***0.54*** | ***0.38-0.77*** | ***<0.001*** |
|  | IL-6 |  | 1.03 | 0.88-1.22 | 0.682 |
| Model 2 |  |  |  |  |  |
|  | Periodontitis | none/mild | Ref |  |  |
|  |  | moderate | 0.96 | 0.69-1.33 | 0.793 |
|  |  | severe | 0.96 | 0.66-1.41 | 0.842 |
|  | ***Age*** |  | ***2.95*** | ***2.52-3.45*** | ***<0.001*** |
|  | ***Sex*** | ***Men*** | ***Ref*** |  |  |
|  |  | ***Women*** | ***0.60*** | ***0.47-0.78*** | ***<0.001*** |
|  | hs-CRP |  | 1.00 | 0.90-1.11 | 0.994 |
| Model 3 |  |  |  |  |  |
|  | Periodontitis | none/mild | Ref |  |  |
|  |  | moderate | 0.90 | 0.57-1.42 | 0.64274 |
|  |  | severe | 0.84 | 0.49-1.44 | 0.52235 |
|  | ***Age*** |  | ***2.70*** | ***2.17-3.38*** | ***< 0.001*** |
|  | ***Sex*** | ***Men*** | ***Ref*** |  |  |
|  |  | ***Women*** | ***0.55*** | ***0.39-0.79*** | ***0.00125*** |
|  | IL-6 |  | 1.04 | 0.87-1.26 | 0.64485 |
|  | hs-CRP |  | 1.00 | 0.78-1.27 | 0.98895 |

N=6,209 participants; 561 cases of AF. Italics bold: p<0.05

**Comment 3: In addition, I do not think reference 25 would be a suitable to cite since these studies are merely based upon insurance claims, not clinical data.
Reply:** It is true that ref. 25 is based on insurance claims. We cited it because it is a massive study; but you are right that it may not be suitable to cite. We removed it from the references in the revised manuscript.

We also ensured that the manuscript meets the PLOS ONE's style, included the tables as part of the main manuscript, and amended the ‘Funding Information’ and ‘Financial Disclosure’ sections. Please, also note the updated invoice address for payment of the PLOS ONE publication fee (Prof. Renate Schnabel, University Medical Center Hamburg Eppendorf, Clinic for Cardiology, Martinistr. 52, 20246 Hamburg, Germany, Email: r.schnabel@uke.de).

Thank you once more for the opportunity to revise our manuscript.

With kind regards

Prof. Dr. Udo Seedorf

**Supporting references**

1.	Wyczalkowska-Tomasik A, Czarkowska-Paczek B, Zielenkiewicz M, Paczek L. Inflammatory Markers Change with Age, but do not Fall Beyond Reported Normal Ranges. Arch Immunol Ther Exp (Warsz). 2016;64(3):249-54. Epub 2015/08/18. doi: 10.1007/s00005-015-0357-7. PubMed PMID: 26283530; PubMed Central PMCID: PMCPMC4863028.

2.	Khera A, McGuire DK, Murphy SA, Stanek HG, Das SR, Vongpatanasin W, et al. Race and gender differences in C-reactive protein levels. J Am Coll Cardiol. 2005;46(3):464-9. doi: 10.1016/j.jacc.2005.04.051. PubMed PMID: 16053959.
