# Supplementary material for: Periodontitis, dental plaque, and atrial fibrillation in the Hamburg City Health Study
Source: PLoS One. 2021 Nov 22;16(11):e0259652. doi: 10.1371/journal.pone.0259652 (PMC8608306; doi:10.1371/journal.pone.0259652)
Supplement: Attachment — Submitted filename: Reply_to_Reviewers_comments_rev1.docx [file pone.0259652.s009.docx]

6. Review Comments to the Author

Reviewer #1:

It would be useful if the authors shared data on how many patients were already diagnosed with AF, and how many they tested for AF who were diagnosed with unknown AF.

**Rely: Of the 561 AF cases, 521 were already diagnosed with AF and 40 additional cases with previously unknown AF were newly identified in the study by the ECGs. The total number of ECGs was 9,087. We added a sampling flow chart to the supplementary material to better characterize the sample.**

If a patient was already diagnosed with AF in the medical was this information known to the researcher prior to the plaque index being carried out?

**Reply: No, this was not known.**

There is no cause or effect with this type of study as it is cross-sectional they cannot state oral health preventing AF based on their study.

**Reply: That is true. We removed the statement from the conclusions.**

If there was an exclusion criteria for those who already had AF then can this be stated clearly in the methods

**Reply: No, this was not an exclusion criterion. This is a cross-sectional study looking at prevalent AF and prevalent PD at baseline with PD as independent variable and AF as dependent variable.**

According to a recently published systematic review, only two small clinical studies had been looked at a potential association between PD and AF over the last fifty years [22].
Incorrect, this SR states two small clinical studies looking at acute infections, not PD, also here is a useful and recent citation
Sen S, Redd K, Trivedi T, Moss K, Alonso A, Soliman EZ, Magnani JW, Chen LY, Gottesman RF, Rosamond W, Beck J, Offenbacher S. Periodontal Disease, Atrial Fibrillation and Stroke. Am Heart J. 2021 May;235:36-43. doi: 10.1016/j.ahj.2021.01.009. Epub 2021 Jan 24. PMID: 33503409; PMCID: PMC8084947.

**Reply: Thank you. We discuss this excellent recently published work in the discussion of the revised manuscript.**

The examination was carried out by trained and certified study nurses.
Normally dentists are required to examine patients and diagnose PD, not nurses- if nurses then is diagnosis inaccurate? If nurses are trained then how are they trained to ensure accurate diagnosis are they performing the periodontal charts? more detail needed here.

**Reply: The term “study nurse”, which is used here, refers to extensively trained and calibrated staff, including advanced dentistry students performing their DDS/DMD doctoral thesis work, especially trained dentist’s assistants, and other medical assistants with extensive experience in conducting the examination, which was performed according to a pre-specified SOP under the supervision of a certified dentist. The “study nurses” did not “diagnose periodontitis”, as you say. They collected the raw data, such as number of teeth, pocket depths, number of bleeding points on probing etc., which were then used by two certified dentists to establish the diagnosis. Data accuracy was established by training and calibration of the staff, electronic data capture and transfer, longitudinal performance evaluation, and statistical monitoring. The Kappa statistics for the tooth count assessment ranged from 0.96 to 1.00, for untreated dental caries Kappa scores were 0.93 to 1.00. The overall Kappa statistics for identifying more complex traits, such as moderate and severe periodontitis according to the CDC/AAP case definition, ranged between 0.60 and 0.70, which was comparable with published results by Dye et al. BMC Oral Health (2019) 19:95 for NHANES. Thus, we are confident that the data are accurate. On the other hand, the high fraction of missing data, which was due to a shortage of sufficiently well trained staff, should be noted as a disadvantage. We provided more details in the methods section and added a sampling chart to the supplementary material (Fig. S1).**

Based on the measurements, a calculation of the decayed, missing and filled teeth (DMFT) index was performed and the plaque index (PI) of Silness-Löe (1964) was assessed
Would have liked to see more information in the methods about systematic and rigourous testing of scores- for example, were the plaque scores assessed by 2 different clinicians, if there was disagreement then would a third clinician be involved

**Reply: All scores that we used are in essence very simple and have been in use in dentistry already for a long time. We provided more details in the methods section.**

Although (DMFT) index is a valid measurement tool it has disadvantages as people can lose teeth from trauma or for orthodontic treatment or tooth decay (eg not just from periodontitis).

**Reply: We agree with you. However, we have not used the DMFT for any evaluations. Nevertheless, the DMFT may be interesting for some readers because it is a widely used indicator of overall dental health. The actual values show that our cohort in fact has a very good oral health.**

The diagnosis of PD and the classification of severity were performed based on the criteria of Eke and Page [26]:
26. Chen SJ, Liu CJ, Chao TF, Wang KL, Chen TJ, Chou P, et al. Dental scaling and atrial fibrillation: a 241 nationwide cohort study. International journal of cardiology. 2013;168(3):2300-3. doi: 242 10.1016/j.ijcard.2013.01.192. PubMed PMID: 23453452.
Incorrect citation placed.

**Reply: Thank you. We corrected this error.**

Assessment of cardiac variables
Authors have used a combined method of diagnosing for AF including self reporting and medical investigations with 24 hour ECG- 1) self reporting is considered inaccurate for diagnosing AF, diagnosing all patients medically would have been more accurate

**Reply: Paroxysmal and some persistent forms of AF are difficult to detect by a single ECG examination in an epidemiological study. Therefore, we relied also on the medical history to identify such cases. People are usually aware of their diagnosis and about ¼ of participants who reported a history of AF could actually be verified by ECG. In contrast, only 0.4% of those who reported no history of AF presented with AF according to the ECG. This shows that verifiable cases were highly enriched in the self-reporting group. Nevertheless you are right that self-reporting may lead to some miss-classification. On the other hand, we would have missed some cases of paroxysmal or persistent AF, if we had decided to not consider the reports. We added this consideration to the discussion section.**

would like to see a flowchart of patient journey and data which would make the results of the research more easily accessible and clear

**Reply: Very good suggestion. We added a flowchart to the supplementary material.**

adjusted for all confounders considered relevant (age, sex, body mass index, diabetes, smoking, and hs-CRP)
There are many more confounders considered relevant- please amend statement to adjusted for some confounders considered relevant

**Reply: Okay, done.**

Figure 1 and figure 2 are not clearly labelled, figure 1 no label of unit measurement for y axis is it a percentage, figure 2 for me was not absolutely clear what the units of measurements were as the bar charts had numbers on the top but I do not know what they represented.

**Reply: Please, see improved figures in the revised manuscript.**

Reviewer #2:

Could the authors explain the rational for using the CHARGE-AF score to decide if patients where having a 24-hrs ECG monitoring or not in the methods section.

**Reply: This was originally planned according to the study protocol in order to identify cases within the high risk group that did not show up in the 12-lead resting ECGs (i.e. paroxysmal, persistent AF). However, the devices were not well tolerated by the participants and only 330 participants received them. No additional AF cases could be identified by the 24-hrs ECGs. Thus, this strategy was cancelled early on in the study and CHARGE-AF scores were not determined systematically. We amended the text in methods section accordingly.**

Add the CHARGE-AF score for each group of PD as a part of the results.

**Reply: The scores are available only for a small fraction recruited at the very beginning. Thus, we refrain from reporting them.**

The authors include the LVEF in different groups of PD as a part of their results. Do they have information of other echocardiographic parameters? It would be particularly interesting if they provide data on left atrium (volume, diameter), since it is a marker of FA risk.

**Reply: These data are not yet fully quality controlled and could therefore not be used.**

This is a suggestion: Most of the data that shows an association between PD and cardiac structural and functional abnormalities detected with echocardiogram (mass, myocardial deformation), comes from studies in patients with diabetes mellitus, hypertension and chronic kidney disease. In all of these studies, there was a clear association between PD severity and left ventricular hypertrophy and abnormal ventricular function. (You can see: Periodontal Disease, Systemic Inflammation and the Risk of Cardiovascular Disease. Heart Lung Circ. 2018 Nov;27(11):1327-1334, for a description of some of these studies). In our laboratory, we have done some work in otherwise healthy subjects with different grades of PD using echocardiography, and we could not find an association between PD and LVH or abnormalities in LV function. Although the reasons can be diverse, there is a possibility that PD and the systemic inflammatory process that comes with it acts as a boost for CVD; perhaps, PD might not be enough to cause CV abnormalities, but when added to other types of stress to the CV system (Hypertension, DM) it can cause CVD.

**Reply: This is an interesting idea. We are constantly expanding the cohort to increase the case numbers and thus the study’s power. At present, the power is still not high enough for informative sub-group analyses of the kind you are suggesting.**

Since AF is multifactorial, and abnormalities in cardiac structure and function can play a role, this can be important for your study. More than 50 of your study population have hypertension; perhaps, you can consider a last analysis including only those with HTN and divide them in those with none/mild PD and severe PD to look for an association between AF and PD, in unadjusted and adjusted analysis.

**Reply: We did this. Please, see Results section and supplementary material.**

Reviewer #3:

Recent results from ARIC cohort study on PD-AF association not discussed

**Reply: Okay, fixed.**

DMFT assessed, a measure primarily for Caries. Data not used for analysis

**Reply: We have not used the DMFT for any evaluations because teeth loss can result from various issues, such as trauma, orthodontic treatment or tooth decay. Nevertheless, the DMFT may be interesting for some readers because it is a widely used indicator of overall dental health. The actual values show that our cohort in fact has a very good overall oral health.**

CRP and IL-6 are potential mediators, hence should not be adjusted for

**Reply: Very good point. We performed a mediation analysis for CRP and IL-6. Please see Results section and supplementary material.**

Socioeconomic status is a potential confounder that is not assessed

**Reply: Thank you, that is true. We included educational level in the revision as a proxy for the SES (It is difficult to obtain correct income information in surveys, which renders the SES unreliable).**

Based on hypothesis oral care should be associated with less AF

**Reply: The prevalence of AF was lower in participants brushing their teeth 2x and more compared with those brushing less than 2 times daily.**

Assessment of AF using questionnaire not a validated method

**Reply: Paroxysmal and some persistent forms of AF, which account for ~50% of all cases of AF, are difficult to detect by a single ECG examination in an epidemiological study. Therefore, we relied also on the medical history to identify such cases. People are usually aware of their diagnosis and about ¼ of participants who reported a history of AF could actually be verified by ECG in our study. In contrast, only 0.4% of those who reported no history of AF presented with AF according to the ECG. This shows that verifiable cases were highly enriched in the self-reporting group. Nevertheless you are right that self-reporting may have led to some miss-classification. On the other hand, we would have missed most cases of paroxysmal or persistent AF, if we had not included self-reported AF. We added this consideration to the discussion section.**
