# Supplementary material for: Periodontitis, dental plaque, and atrial fibrillation in the Hamburg City Health Study
Source: PLoS One. 2021 Nov 22;16(11):e0259652. doi: 10.1371/journal.pone.0259652 (PMC8608306; doi:10.1371/journal.pone.0259652)
Supplement: Attachment — Submitted filename: Response to Reviewers_rev2.docx [file pone.0259652.s010.docx]

### PONE-D-21-08970R2

**Response to Reviewers**

6. Review Comments to the Author

Please use the space provided to explain your answers to the questions above. You may also include additional comments for the author, including concerns about dual publication, research ethics, or publication ethics. (Please upload your review as an attachment if it exceeds 20,000 characters)

Reviewer #1: No further suggestions as the author has responded to all points and improved the overall validity of the article

**Reply: Thank you very much.**

Reviewer #2: The authors have made a great effort addressing the reviewers’ suggestions; the quality of the manuscript has improved.

Although the overall results of the study do not suggest an association between PD and AF, the study is still of relevance since it adds valuable information to the field, I think the paper is now ready to be considered for publication.

**Reply: Many thanks.**

Reviewer #3: The reviewers comments have been addressed. I would suggest following changes:

1) Abstract conclusion starts with "no finding" first, and readers have to go sentences down to figure out what the positive findings of PI -->AF association

**Reply: Thank you for this suggestion. We changed the sequence in the Abstract conclusions, which now reads: “The study shows an association between prevalent AF and increased dental plaque levels indicated by a higher PI. In contrast, an association of prevalent PD with prevalent AF after adjustments for several confounders could not be demonstrated.”**

2) DMFT is an useful marker of oral health and dental caries. If the DMFT score is better in the group, please indicate that as a reason why it was not tested for association with AF. Alternately please include the analysis for readers to know that DMFT is not associated with AF

**Reply: Thank you very much for this comment. We added the analysis concerning the DMFT to the Result section (please, see new Table 4, which shows that the DMFT is not associated with AF after adjustment for age and sex). We also mention this result in the Abstract and the Discussion (please see manuscript with track changes).**
